# The efficacy and safety of herbal medicine BHH10 in postmenopausal women with osteoporosis: study protocol for a phase II, multicenter, randomized, double-blinded, placebo-controlled clinical trial

**DOI:** 10.1186/s13063-018-2854-6

**Published:** 2018-09-10

**Authors:** Yeeun Cho, Seunghoon Lee, Jihye Kim, Jung Won Kang, Yong-Hyeon Baek, Byung-Kwan Seo, Jae-Dong Lee

**Affiliations:** 1 Department of Clinical Korean Medicine, Graduate School, Kyung Hee University, Seoul, South Korea; 2 Department of Acupuncture & Moxibustion Medicine, Kyung Hee University Hospital of Korean Medicine, Seoul, 02447 South Korea; 3 Department of Acupuncture & Moxibustion, College of Korean Medicine, Kyung Hee University, Seoul, South Korea; 4 Oriental Medicine Research Center for Bone & Joint Disease, East-West Bone & Joint Research Institute, Kyung Hee University, Seoul, South Korea

**Keywords:** Osteoporosis, Postmenopausal women, Herbal medicine, BHH10, Protocol

## Abstract

**Background:**

Osteoporosis is becoming more prevalent in aging societies worldwide, and the economic burden attributable to osteoporotic fractures is substantial. The medications presently available to treat osteoporosis have side effects, and the development of safer and more effective treatments is urgently needed. The aim of this study is to evaluate the efficacy and safety of BHH10, a traditional Korean herbal medicine, in the treatment of postmenopausal osteoporosis.

**Methods/Design:**

This is a phase II, multicenter, randomized, double-blinded, placebo-controlled clinical trial and will include 168 postmenopausal women aged 55 years and older with osteoporosis. The participants will be recruited competitively from two sites of the Acupuncture and Moxibustion Department of Kyung Hee University Hospital of Korean Medicine, either Hoegidong or Gangdong in Seoul, Korea. Participants will be assigned randomly to one of two groups, the BHH10 group or the placebo group, in a 1:1 ratio, and will have five scheduled visits. Participants will take two tablets of BHH10 or placebo three times daily for 12 weeks. The primary efficacy outcome is the change in bone mineral density at the lumbar spine (L1–4) between baseline (visit 1) and 12 weeks after randomization (visit 5). Other outcome variables include changes in bone turnover markers, the Deficiency Syndrome of the Kidney Index, EuroQol five-dimension questionnaire score, and laboratory parameters, as well as adverse events.

**Discussion:**

To our knowledge, this will be the first clinical trial to assess the efficacy and safety of BHH10 in postmenopausal women with osteoporosis. It is anticipated that the results will contribute to the development of traditional herbal medicines that can be used to treat osteoporosis in postmenopausal women in Korea. If the superiority of BHH10 over placebo is demonstrated, this study could provide the foundation for a phase III clinical trial. The results of the study will be published in a peer-reviewed journal.

**Trial registration:**

Clinical Research Information Service, KCT0001842. Registered on 14 March 2016.

**Electronic supplementary material:**

The online version of this article (10.1186/s13063-018-2854-6) contains supplementary material, which is available to authorized users.

## Background

Osteoporosis is a systemic skeletal disease characterized by decreased bone mass and altered microstructure that leads to bone fragility and an increased risk of fractures [1]. As osteoporosis is easily overlooked due to the lack of subjective symptoms, the prevalence of osteoporosis is higher in aged people. In 2010, more than 99 million individuals aged 50 years or older in the U.S. alone were estimated to have osteoporosis or low bone mass, and this number is projected to increase by 19% by 2020 and by 32% by 2030 [2]. The data sharing statements are detailed in Table 1.

**Table 1:** Data sharing statements

ICMJE requirements	Answer
Will individual participant data be available (including data dictionaries)?	Yes
What data in particular will be shared?	Individual participant data behind the results reported in this article, after deidentification (text, tables, figures, and appendices)
What other documents will be available?	Study protocol
When will data be available (start and end dates)?	Beginning 3 months and ending 5 years following publication of the research
With whom?	Investigators whose proposed use of the data has been approved by an independent review committee identified for this purpose
For what types of analyses?	To achieve the aims in the approved proposal.
By what mechanism will data be made available?	Proposals should be directed to kmdyeeun@gmail.com; to be given access, data requestors must sign a data access agreement

The prevalence of osteoporosis is particularly high in women after the menopause. Approximately 80% of patients with osteoporosis are female and postmenopausal. The hormonal changes stimulate ligands of the receptor activator of nuclear factor-κβ to promote osteoclastogenesis [3]. The fifth Korean National Health and Nutrition Examination Survey in 2010 reported a high risk of osteoporotic fracture in 37.7% of postmenopausal women aged 50 years or older [4]. Aggregated data for women aged 55 years or older from 2000 to 2011 in the U.S. show that the hospitalization costs for osteoporotic fractures are greater than those for myocardial infarctions, breast cancer, or strokes [5].

Many pharmacological agents, such as bisphosphonates, alendronate, risedronate, and denosumab, are used to treat postmenopausal osteoporosis. However, the agents most commonly used are inconvenient to take and compliance is often poor [6]. For example, an oral bisphosphonate tablet must be taken in the morning before breakfast with plenty of water and the patients must remain seated for 30 to 60 min afterwards, and not eat during this time [7]. Furthermore, many patients stop taking the medication because of concerns about side effects such as atypical femoral fractures, osteonecrosis of the jaw, and irritation of the gastrointestinal tract [8].

There is an increasing need for agents that are safer and more effective for the treatment of osteoporosis. According to traditional Korean medicine, kidney dysfunction can cause osteoporosis by inhibiting bone metabolism and decreasing the level of estrogen [9]. Herbal medicines targeted at the kidney, such as *jasinhwan*, are already used to treat osteoporosis [10]. BHH10 is an herbal medicine derived from *jasinhwan* and consists of the roots of *Astragalus membranaceus* (Mongolian milkvetch), twigs of *Cinnamomum cassia* (Chinese cassia or Chinese cinnamon), and the bark of *Phellodendron amurense* (Amur cork tree). BHH10 has been shown to regulate bone resorption and improve bone mineral density (BMD) without toxic effects in ovariectomized rats [11].

This placebo-controlled study is evaluating the efficacy and safety of BHH10 in the treatment of postmenopausal osteoporosis by measuring changes in BMD, levels of bone turnover markers, the Deficiency Syndrome of the Kidney Index (DSKI), and the EuroQol five-dimension questionnaire (EQ-5D) score, as well as adverse events.

The study protocol was developed in accordance with the 2013 Standard Protocol Items: Recommendations for Interventional Trials (Additional file 1).

## Methods/Design

### Objective

The primary aim of this study is to determine if treatment with BHH10 for 12 weeks results in a greater increase in BMD at L1–4 than those who receive a placebo. The secondary outcomes are:

- any significant differences in femoral BMD, bone turnover marker levels, DSKI, and EQ-5D score in the group receiving BHH10 compared to the group receiving the placebo after 12 weeks of treatment
- to determine if there is a causal relationship between the primary outcome and the DSKI or EQ-5D score
- whether BHH10 is a safe treatment based on laboratory parameters, vital signs, and electrocardiography (ECG).

### Design and setting

This study is a phase II multicenter, randomized, placebo-controlled clinical trial with two parallel groups. Participants who voluntarily sign informed consent and are eligible for this study will be assigned randomly to one of two groups (the BHH10 or the placebo group) in a 1:1 allocation. Patients will take two tablets of BHH10 or placebo three times daily for 12 weeks.

#### Recruitment strategy

The study participants were recruited competitively from two sites of the Acupuncture and Moxibustion Department of Kyung Hee University Hospital of Korean Medicine, either Hoegidong or Gangdong in Seoul, Korea. A total of 168 postmenopausal women with osteoporosis were recruited via broadcast media, newspapers, posters, and the Internet homepages of the participating hospitals, as well as advertisements placed in nearby welfare centers. Recruitment started in March 2016 and the accrual target was reached in April 2018.

#### Study plan

**Fig. 1:**
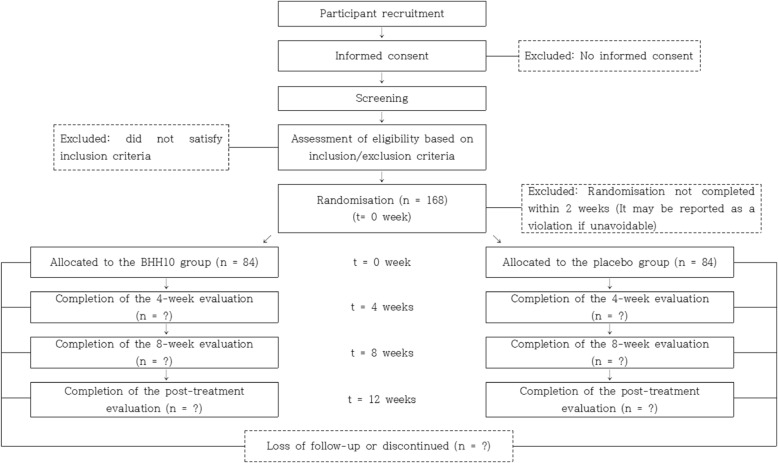
Flow chart of the study

This study will be conducted in two phases (Fig. 1):

1. Screening (2 weeks): Trained researchers in the hospital will explain this trial to and seek written informed consent from potentially eligible patients. Patients who agree to participate in the study will undergo BMD measurements, laboratory investigations, and an ECG. The results of these tests will be conveyed by telephone and patients who meet the inclusion criteria will be randomized within 2 weeks after the screening visit.
2. Treatment (12 weeks): On the second visit, patients will be randomly assigned to one of the two groups (the BHH10 group or the placebo group) and supplied with their allocated investigational drugs after completing the DSKI and EQ-5D questionnaires. Participants will take two tablets of BHH10 or placebo three times daily for 12 weeks. Additional visits will be made at monthly intervals during the 12 weeks of treatment. A total of four visits will be made during the treatment period. A blood test will be conducted 8 weeks after randomization (visit 4). BMD measurements, blood and urine tests, and an ECG will be performed 12 weeks after randomization (visit 5).

### Types of participant

#### Inclusion criteria

Participants must be women who meet the following criteria:

1. Ambulatory and aged 55–85 years
2. Postmenopausal (at least 12 months since the last menstrual period)
3. BMD should be below −2.5 standard deviations
4. Willing to participate and sign a consent form after being given a detailed explanation of the trial

#### Exclusion criteria

Participants will be excluded if any of the following conditions are satisfied:

1. Alkaline phosphatase more than twice the upper limit of normal
2. Liver disease (aspartate transaminase or alanine transaminase more than twice the upper limit of normal)
3. Kidney disease (creatinine > 2.0 mg/dL)
4. Hypercalcemia (calcium > 10.5 mg/dL)
5. Uncontrolled chronic disorders that could affect bone metabolism (liver disease, alcoholism, primary hyperparathyroidism, or malignancy)
6. Pharmacotherapy for osteoporosis (except calcium or vitamin D) in the previous 3 months
7. Injection of an agent for osteoporosis in the previous 6 months
8. Systemic administration of an agent that may affect calcium metabolism in bone, such as a systemic corticosteroid or diuretic, for more than 6 months (subjects who have not received forbidden drugs except contraceptive injections within the previous 3 months before screening may be eligible to participate)
9. Metabolic bone diseases other than osteoporosis
10. A mental disorder likely to affect compliance with the requirements of the trial

### Randomization and allocation concealment

For randomization, an independent blinded statistician will generate random numbers using the SAS randomization program (version 9.4; SAS Institute, Inc., Cary, NC, USA). The manufacturer will supply the hospital with investigational drugs in consecutively numbered drug containers with identical packaging to conceal treatment allocation. Blinded researchers in each hospital will enroll and assign participants to one of the two study groups.

### Blinding

The participants, outcome assessors, study monitors, data managers, and statisticians will be blinded to treatment allocation. If possible, the study participants will be advised to avoid discussing the investigational drugs with other participants. Blinding will be maintained until the 168 participants have completed the study. The success of double-blinding will be assessed at the final visit. The database will then be locked.

An emergency code for each individual has been provided to study researchers and will be disclosed only if it must be known whether a patient is receiving BHH10 or the placebo to manage a serious adverse event.

### Intervention

**Fig. 2:**
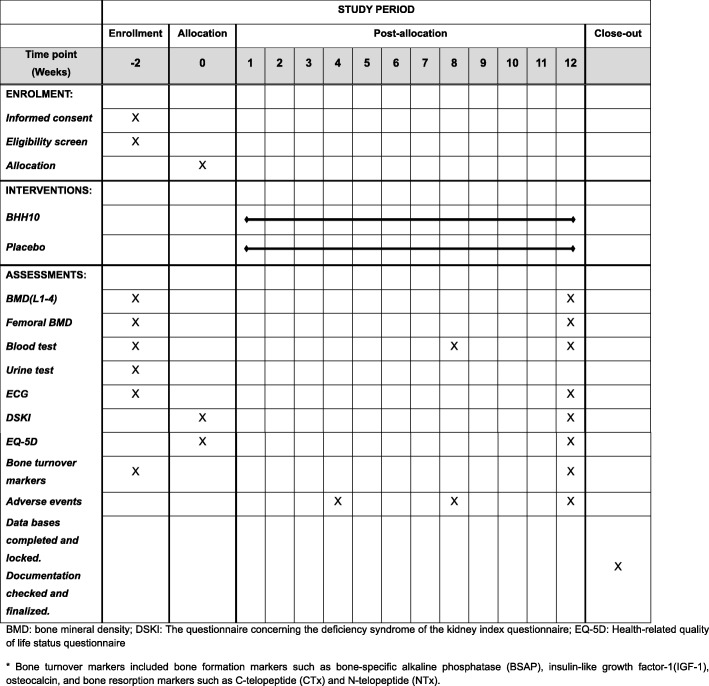
Appearance of investigational drug (BHH10)

The total dose of each tablet of BHH10 is 800 mg, of which 400 mg is 30% ethanol herbal extract (Fig. 2). The herbal extract was obtained from the roots of *A. membranaceus*, the twigs of *C. cassia*, and the bark of *P. amurense* in a ratio of 2:2:1. All plant materials and the samples were deposited in the herbarium of Quality Control of the Hanpoong Pharm and Foods Co., Ltd. (Junju, South Korea). The raw materials were extracted twice with 30% ethanol and vacuum-dried to produce the BHH10 extract (yield, 9.72%).

A high-performance liquid chromatography analysis was performed to quantify the chemical constituents of the BHH10 herbal extract. The herbal extract was controlled to contain more than 1.15 mg/g of cinnamic acid, 0.26 mg/g of formononetin, and 10.67 mg/g of berberine. The purity test, including heavy metal tests, residual pesticide tests, and a microbial limit test, was conducted according to the Korea Pharmacopeia. The extract is tested for the presence of heavy metals, lead, arsenic, DDT, benzene hexachloride aldrin, dieldrin, endrin, total number of aerobic microorganisms, and microorganism counts (including fungi, *Escherichia coli*, *Salmonella*, *Pseudomonas aeruginosa*, and *Staphylococcus aureus*).

**Fig. 3:**
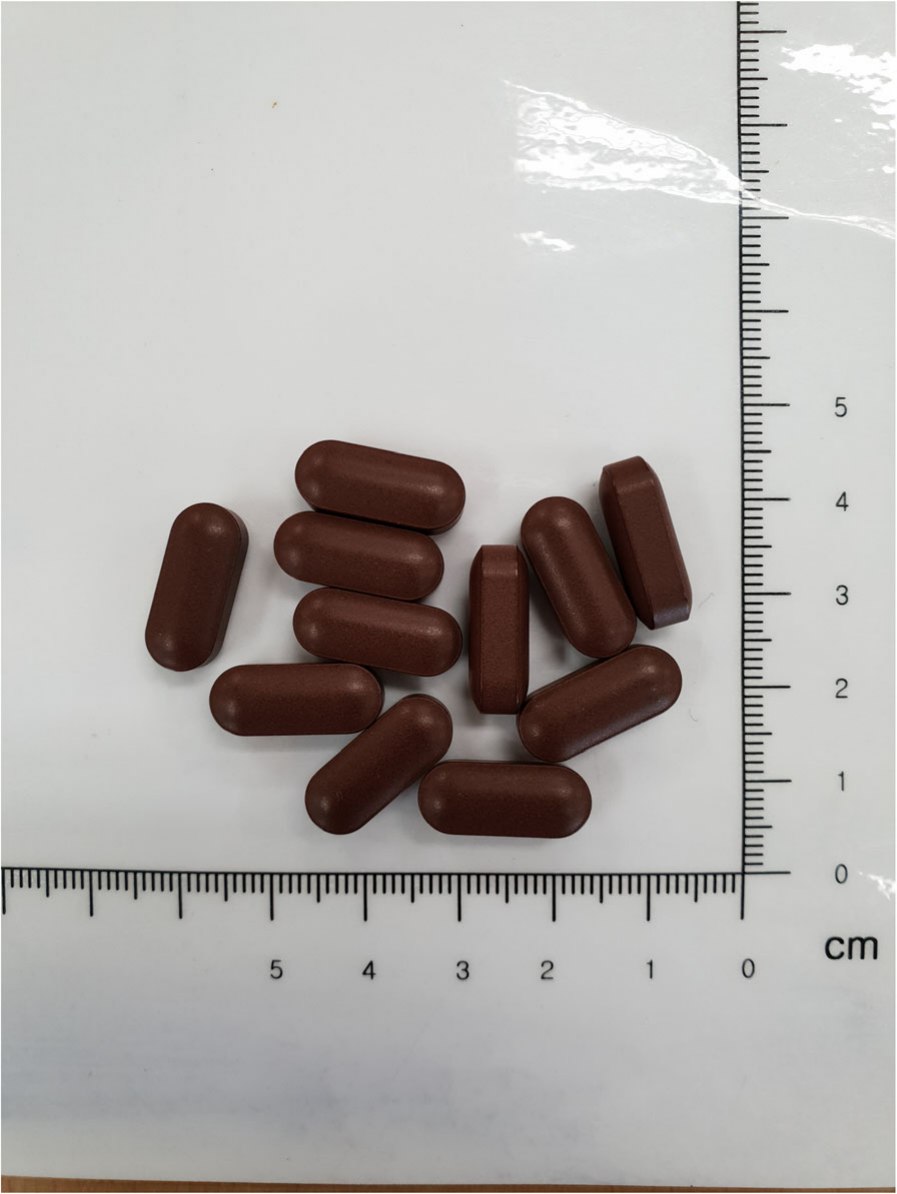
Appearance of investigational drug (placebo)

The placebo tablets contain cellulose, corn starch, diluting agents, and food coloring to match the BHH10 tablets (Fig. 3). The BHH10 and placebo tablets are manufactured to be identical in appearance in terms of shape, color, size, smell, and taste. The BHH10 and placebo tablets are manufactured at Hanpoong Pharm and Foods Co., Ltd. in accordance with Korean Good Manufacturing Practices.

The study participants will take two tablets of BHH10 or placebo, three times daily for 12 weeks. The dosage was determined by reference to preclinical studies [11, 12].

During the 12 weeks of treatment, participants will comply with the study protocol by visiting the clinic every month and completing their scheduled examinations. Unused tablets will be returned and counted at visits 3, 4, and 5. If a participant is unable to take the allocated investigational drug because of serious adverse event, the researchers can alter the dose or frequency according to internal guidelines.

#### Concomitant and forbidden treatments

Drugs that have been taken for an indication other than osteoporosis for more than 30 days before the screening visit may be continued during the study period. Continuation of the same doses of health functional foods, such as calcium or vitamin D, are permitted in this trial only if they have been taken continuously in the 3 months before the screening visit. Prohibited drugs include agents that can affect BMD, such as other medications for osteoporosis, steroids, carbamazepine, phenytoin, phenobarbital, heparin, warfarin, thyroid hormones, gonadotropin-releasing hormone agonists, depot-medroxyprogesterone acetate, anticancer drugs, cyclosporine A, antidepressants, aluminum-containing antacids, aromatase inhibitors, anti-tuberculosis drugs, thiazolidinedione, proton pump inhibitors, antiretroviral drugs, and thiazide diuretics. Participants who have taken or need to take any of these medications will be withdrawn from the study.

### Outcomes

**Fig. 4:**
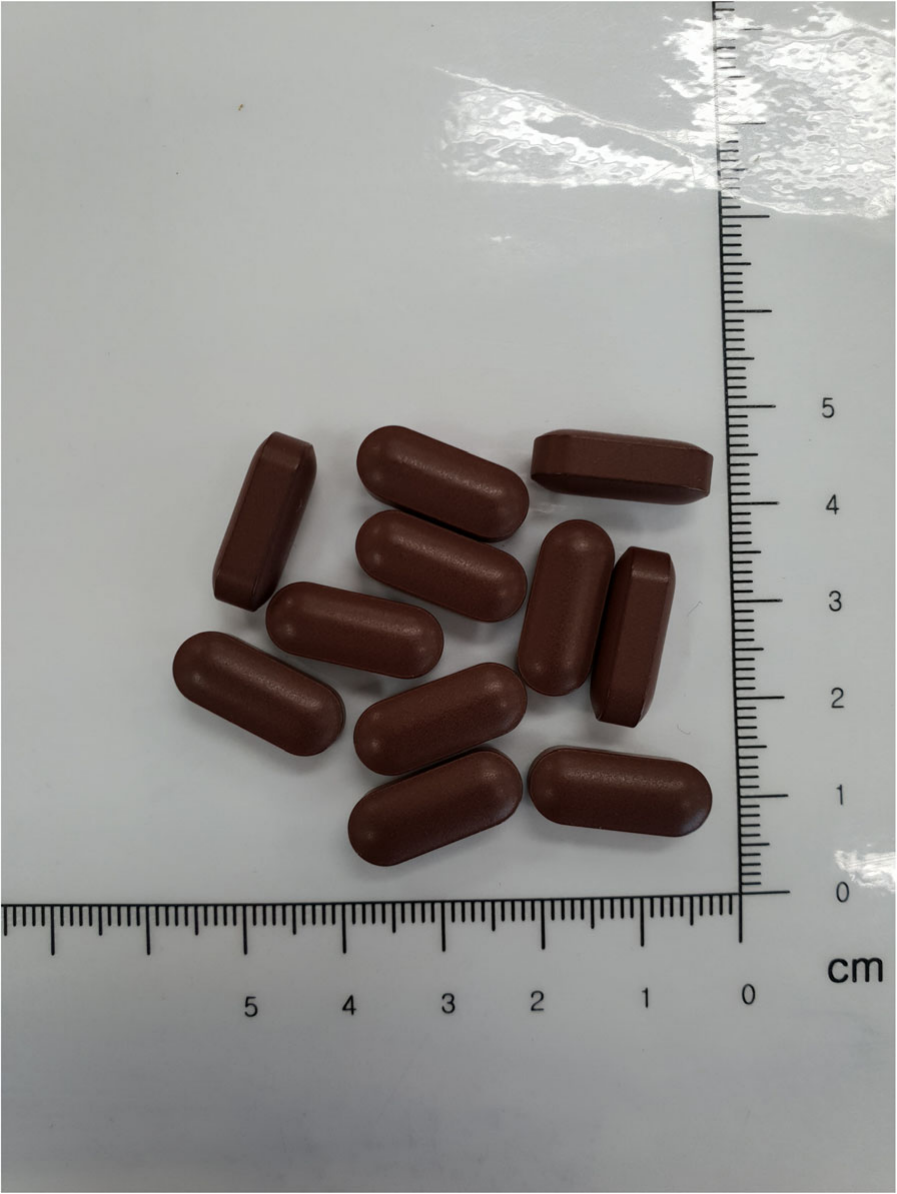
Standard Protocol Items: Recommendations for Interventional Trials (SPIRIT) schedule of enrollment,interventions, and assessments

The measurement points of each evaluation index are shown in Fig. 4.

#### Primary outcome measurement

The primary outcome will be the change in BMD at the lumbar spine (L1–4) between baseline (visit 1) and 12 weeks after randomization (visit 5). BMD will be examined by dual-energy X-ray absorptiometry. Both participating institutions use the same type of X-ray machine (Lunar Prodigy, GE Medical Systems Lunar, Madison, WI, USA).

#### Secondary outcome measurements

##### Femoral BMD

The change in femoral BMD between at baseline (visit 1) and after treatment (visit 5) will be calculated using the lowest values at the femoral neck and total hip.

##### DSKI questionnaire score

The DSKI consists of 12 items examining the deficiency syndrome of the kidney [13]. Each item is rated on a three-point symptom scale (0 none, 1 average, and 2 severe) and the total score is used. The change of the DSKI score between randomization (visit 2) and after treatment (visit 5) will be calculated.

### Health-related quality of life status questionnaire

The EQ-5D is an outcome measurement that is widely used to assess quality of life in patients with osteoporosis [14], and is being used in this trial. This tool contains a five-item description section and a visual analog scale (EQ-VAS) [15]. The five-item description section includes questions on mobility, self-care, usual activity, pain and discomfort, and anxiety and depression. There are three possible responses to each item (no problems, some problems, and severe problems). The participants indicate their present health status by checking the most appropriate score for each of the five items. The EQ-VAS is a self-recorded health score for which the best imaginable health condition is 100 points and the worst is 0 points. The change in the score for each item of the EQ-5D between randomization (visit 2) and after treatment (visit 5) will be measured.

### Bone turnover markers

Levels of bone formation markers (bone-specific alkaline phosphatase, insulin-like growth factor-1, and osteocalcin) and bone resorption markers (C-telopeptide and N-telopeptide) will be observed at baseline (visit 1) and after treatment (visit 5). The change in these markers will be measured.

### Sample size

The primary null hypothesis of this study is that there is no difference in the change in BMD at the lumbar spine (L1–4) between the BHH10 and placebo groups after 12 weeks of treatment. In view of the results of a similar study of herbal medicine in postmenopausal women with osteoporosis [16], a mean difference of 0.047 ± 0.097 between the experimental group and control group was assumed. With a 5% significance level and a power of 80%, it was calculated that a sample size of 66 patients per group would be necessary. Allowing for a dropout rate of 20%, a sample size of 168 participants (84 in each group) has been included.

### Criteria for withdrawal

1. Violation of inclusion or exclusion criteria
2. An adverse event or a serious adverse event
3. An acute reaction (allergy or anaphylaxis) to the investigational drug
4. Systemic disease not detected at the time of screening
5. Less than 70% compliance with the investigational treatment at the final evaluation
6. Unsatisfactory treatment effect during the trial
7. Taking of other medication that could affect the results of the study without consulting the researchers
8. Non-compliance with the requirements of the trial
9. Loss to follow-up
10. Worsening of disease or accidental concomitant disease
11. Continuation in the trial is deemed inappropriate by the researchers

### Data management

Researchers who have read and understood the standard operating procedures will obtain written consent and collect and manage the study data from the participants, who will be given ongoing encouragement to complete the study. They are telephoned in advanced of each visit and the importance of taking the investigational drugs at the correct times is reinforced. If a participant needs to be withdrawn from the study, the scheduled tests are still performed at the final study visit if the participant is agreeable. If a follow-up investigation is required, researchers will contact the participant by telephone as necessary. After the end of the trial, data entry will be by double entry, and matching will be conducted after inconsistent data has been reviewed. When the data are matched, a data clarification form will be completed and validated. The resolution will be reflected in the data, and medical coding will be performed using MedDRA (version 17.0).

### Statistical analysis

The analysis data set will consist of an intention-to-treat data set, a per protocol (PP) data set, and a safety data set. The intention-to-treat data set will include all subjects assigned to each group. The PP data set will include only participants who adhered to the study protocol and completed the clinical study. The minimum compliance rate for participants taking the investigational drugs in the PP data set is 80%. The safety data set will include any participants who were randomly assigned to and received at least one dose of the investigational drug. The intention-to-treat analysis will be the primary analysis and will be compared with the PP analysis in a sensitivity analysis. The last observation carried forward method will be used to manage missing data.

For the descriptive analysis, the two-sample Student’s *t*-test or Wilcoxon rank sum test will be used for continuous data and the chi-squared test or Fisher’s exact test will be used for categorical data depending on whether the data are normally distributed or skewed.

For the confirmatory analysis, the primary efficacy endpoint, the change in BMD at the lumbar spine (L1–4) between baseline and 12 weeks, will be evaluated using Student’s *t*-test or the Wilcoxon rank sum test. The secondary endpoints will be evaluated using Student’s *t*-test or the Wilcoxon rank sum test for continuous data or the chi-squared test or Fisher’s exact test for categorical data. We will perform a regression analysis to examine the causal relationship between the DSKI, EQ-5D, and the primary outcome. All statistical analyses will be performed by a statistician blinded to treatment allocation using SAS (version 9.4, SAS Institute Inc., Cary, NC, USA). A significance level of 5% will be used.

### Data monitoring

The clinical research organization, Neoneutra Co., Ltd. (Seoul, South Korea), will conduct regular monitoring to ensure the quality of the data. Its monitors check that the randomly assigned study participants meet the inclusion and exclusion criteria and that the study is proceeding well, and they ensure that the data are adequately recorded in the case report forms. There will be no interim analysis. The study will continue until the 168 participants have completed the study.

### Adverse events

Researchers will record patient-reported symptoms as adverse events on a case report form at each study visit. The causal relationship between the investigational drug and an adverse event will be assessed using a six-point grading scale (1 definitely related, 2 probably related, 3 possibly related, 4 probably not related, 5 definitely not related, and 6 unknown). The severity of the adverse event will be scored on a three-point scale (1 mild, 2 moderate, and 3 severe). If an adverse event is deemed to be caused by the investigational drugs, the dose or frequency can be altered according to the standard operating procedures. There are options for reimbursement through clinical trial insurance if a study participant requires treatment due to an adverse event related to the investigational drugs.

## Discussion

This is the study protocol of a phase II, multicenter, randomized, double-blinded, placebo-controlled trial assessing the effect of BHH10 on postmenopausal women with osteoporosis. This study aims to evaluate the efficacy and safety of BHH10 compared to a placebo after 12 weeks of treatment.

Some clinical studies of the effect of herbal medicine on patients with osteoporosis have been reported. A recent Cochrane systematic review reported that the quality of evidence was generally low and that most included studies (85 of 108) had small sample sizes, ranging from 20 to 120. Therefore, the evidence for the use of herbal medicine in patients with osteoporosis is not conclusive and more rigorously designed studies with larger sample sizes are required [17]. Our study has been designed to take into account the suggestions made for additional research in the Cochrane review. The study will recruit a total of 168 participants to compare the effect of the herbal medicine BHH10 with that of the placebo in postmenopausal women with osteoporosis. The methods used for randomization, treatment concealment, and blinding are at low risk of bias and will be described in detail in the final report. Furthermore, selected biomarkers of bone turnover will be measured to explore the potential advantages of BHH10 in terms of the biochemistry of the bone matrix.

BHH10 includes three herbal medicines: *A. membranaceus*, *C. cassia*, and *P. amurense*. Formononetin, the active ingredient in *A. membranaceus*, has been reported to have a favorable effect on bone formation. One in vitro study reported that formononetin had a positive effect on osteoblasts by increasing the expression of alkaline phosphatase, vascular endothelial growth factor, bone morphogenic protein-2, type I collagen, and osteocalcin in subchondral osteoblasts isolated from normal subjects, and by suppressing their expression in subchondral osteoblasts isolated from patients with arthritis. That study confirmed that formononetin promoted the formation of normal bone cells and inhibited the overexpression of osteogenic genes and cytokines [18]. Furthermore, the *C. cassia* component of BHH10 has been reported to stimulate bone formation in an osteoblastic MC3T3-E1 cell line [19]. Based on the above findings, it is hoped that BHH10 will not only inhibit bone resorption but also promote bone formation.

This trial has some limitations. First, it will not be possible to assess the long-term effects of BHH10 because there will be no follow-up after the 12-week study period. Second, whether the participants take the investigational drugs at the correct time will not be known because no dosage log will be completed.

However, through this rigorous validation, it is hoped that the results of this randomized controlled trial will provide evidence of the efficacy and safety of BHH10 and pave the way for a longer-term confirmatory phase III trial as well as studies of other herbal medicines for postmenopausal women with osteoporosis.

### Trial status

The study was launched on 24 March 2016. The most recent version of the protocol (version 2.7) was approved by the institutional review board on 11 August 2017. Recruitment was completed in April 2018. The results will be published in a peer-reviewed journal.

## Additional file

Additional file 1: SPIRIT 2013 Checklist: Recommended items to address in a clinical trial protocol and related documents. (DOC 131 kb)

## Abbreviations

BMD: Bone mineral density
DSKI: Deficiency Syndrome of the Kidney Index
ECG: Electrocardiography
EQ-5D: EuroQol five-dimension questionnaire
EQ-VAS: EuroQol visual analog scale
PP: Per protocol
SAS: Statistical Analysis System
